# Evaluation of serum ESPL1 as a biomarker for early diagnosis of HBV-related hepatocellular carcinoma

**DOI:** 10.3389/fonc.2025.1574317

**Published:** 2025-04-03

**Authors:** Lu-Huai Feng, Lu Wei, Bobin Hu, Hengkai Liang, Qingmei Li, Qianbing Yin, Tumei Su, Long Huang, Hongqian Liang, Jianning Jiang, Minghua Su

**Affiliations:** ^1^ Department of Infectious Diseases, The First Affiliated Hospital of Guangxi Medical University, Nanning, Guangxi, China; ^2^ Key Laboratory of Early Prevention and Treatment of Regional High Incidence Tumors (Guangxi Medical University), Ministry of Education, Nanning, Guangxi, China

**Keywords:** hepatocellular carcinoma, alpha-fetoprotein, early diagnosis, HBV S gene fusion, biomarker

## Abstract

**Objective:**

To evaluate the reliability of serum human phosphorylated exospindle polar-like proteinase 1 (ESPL1) as a serum biomarker for early diagnosis of hepatitis B virus (HBV)-related hepatocellular carcinoma (HBV-HCC).

**Methods:**

This retrospective study was conducted on 266 patients with chronic hepatitis B (CHB), liver cirrhosis (LC), and HBV-related HCC. Data on demographics and clinical information were collected, and ESPL1 levels were measured using enzyme linked immunosorbent assay. Levels of ESPL1, alpha-fetoprotein (AFP), and protein induced by vitamin K absence -II (PIVKA-II) were compared at different disease stages, and spearman correlation analysis was used to assess their relationship with clinical markers. The diagnostic accuracy of ESPL1, AFP, and PIVKA-II for early HBV- HCC was assessed using ROC curve analysis.

**Results:**

The study comprised 121 patients diagnosed with CHB, 98 patients with LC, and 47 patients with HBV-HCC. Serum ESPL1 levels show an increasing trend across groups with chronic HBV infection, CHB, LC, and HBV-HCC, with levels at 224.6 ng/L, 285.8 ng/L, and 440.4 ng/L (in pairwise comparison, *P*<0.05). Serum AFP and PIVKA-II levels displayed no significant statistical differences between the CHB and LC groups. Spearman correlation analysis revealed that levels of ESPL1, PIVKA-II, and AFP are not influenced by clinical characteristics and show no correlation with each other. ROC curve analysis indicated that the optimal diagnostic threshold for ESPL1 in HBV-HCC is 345.7 ng/L, with AUC values for ESPL1, PIVKA-II, and AFP being 0.797 (95% CI: [0.708-0.886]), 0.788 (95% CI: [0.718-0.858]), and 0.572 (95% CI: [0.523-0.624]). In AFP and PIVKA-II negative patients, the AUC values for ESPL1 diagnosis of HBV-HCC were 0.79 and 0.83.

**Conclusion:**

ESPL1 is a potential biomarker for tracking chronic HBV infection and predicting the development of HBV-HCC. Monitoring ESPL1 levels in serum could help with early detection and personalized screening HBV-HCC for individuals with chronic HBV infection.

## Introduction

According to the World Health Organization, over 200 million people worldwide suffer from chronic HBV infection and approximately 15-25% will develop liver cirrhosis or HBV-related hepatocellular carcinoma (HBV-HCC) during their lifetime. The morbidity and mortality rates of HBV-HCC are expected to rise annually over the next 20 years (1). For patients with HCC who are eligible for surgery, surgical resection offers the best chance for a cure. However, only 10%-30% of HBV-HCC cases undergo curative surgical resection, and the high rate of postoperative recurrence remains a significant threat to patient survival (2). Constrained by the challenge of early diagnosis, numerous patients are compelled to pursue alternative treatments, such as hepatic artery embolization (3). Notably, advancements in molecular targeted drugs and immunotherapy, such as tyrosine kinase inhibitors and immune checkpoint inhibitors, have significantly enhanced patient prognosis (4). Nevertheless, the range of available treatment modalities remains limited. It is imperative to continue investigating novel biomarkers for the early detection of HBV-related hepatocellular carcinoma (5).

In recent years, increasing research has focused on the development of serological markers for the early diagnosis and detection of HBV-HCC, yet effective serum biomarkers for monitoring and early diagnosis remain elusive. AFP is currently the most used serum biomarker for monitoring HBV-HCC. However, the sensitivity of AFP for diagnosing HBV-HCC is only between 25% and 65% and approximately 30%-40% of HBV-HCC patients consistently test negative for serum AFP (6). The heterogenous isoform of AFP (AFP-L3) is considered to have higher specificity in diagnosing HBV-HCC. However, the proportion of serum AFP-L3 is heavily influenced by the levels of AFP (7). Protein induced by PIVKA-II is an abnormal prothrombin produced specifically in association with HBV-HCC, independent of AFP secretion. However, the accuracy of PIVKA-II as a screening tool for HBV-HCC is disputed, primarily due to variations in its expression across different ethnicities and deficiencies in testing technology (7, 8). Notably, recent advancements in the detection of circulating nucleic acid biomarkers, including cell-free DNA (e.g., circulating tumor DNA, whole genome sequencing of cfDNA, methylation/hydroxymethylation of cfDNA, cfDNA gene mutations, and cell-free viral DNA), exosomes, and microRNA, are considered the next generation of biomarkers for the early diagnosis and screening of HBV-HCC. However, due to a lack of clinical validation and high testing costs, their clinical acceptance remains limited. To date, there is no international consensus on an ideal serum biomarker for early diagnosis and screening of HBV-HCC (9). The exploration and development of new biomarkers that accurately reflect the natural history or progression stages of chronic HBV infection, closely monitor disease progression, and enable early detection of HBV-HCC continue to be significant clinical challenges (10).

Previous research conducted by our team has revealed a notable prevalence of the ESPL1 fusion gene, resulting from the integration of the HBV S gene, in individuals diagnosed with HBV-HCC. Our findings indicate that serum levels of ESPL1 may serve as a valuable indicator of the severity of HBV-HCC, facilitating the distinction between small liver cancers and cirrhotic nodules (11–18). Building upon this foundation, the current study aims to investigate the clinical efficacy of serum ESPL1 in comparison to AFP and PIVKA-II for the early detection of HBV-HCC, and determine whether serum ESPL1 levels can be utilized as a novel and specific biomarker for the prompt diagnosis and screening of HBV-HCC.

## Patients and methods

### Patients

From a follow-up cohort of patients infected with HBV who visited the First Affiliated Hospital of Guangxi Medical University between January 2002 and November 2023, a total of 266 individuals with adequate serum samples were selected for the evaluation of serum ESPL1 levels. This study consisted of 121 patients with chronic hepatitis B (CHB), 98 with liver cirrhosis (LC), and 47 with HBV-HCC. Detailed clinical characteristics pertaining to chronic HBV infection, such as age, gender, antiviral treatment regimen, ALT levels, HBeAg status, HBV DNA levels, AFP levels, and PIVKA-II levels, were collected for all participants.

### Inclusion and exclusion criteria

Inclusion Criteria: 1. Study subjects must be chronic HBV infection patients, including those with CHB and HBV-induced cirrhosis. 2. HBV-HCC patients must have tumor diameters less than 3 cm, with a diagnosis conforming to small hepatocellular carcinoma. All participants in this study were categorized as stage 0 or stage A in accordance with the Barcelona Clinic Liver Cancer staging criteria.

Exclusion Criteria: 1. Patients with HBV infection co-infected with Hepatitis D, E, Hepatitis C, HIV, or other infectious diseases. 2. Patients with concurrent non-HCC malignancies. 3. Patients with liver diseases caused by other factors, such as liver fluke infection, alcoholic liver disease, fatty liver disease, or autoimmune liver diseases.

### Serum sample collection and storage

Peripheral venous blood samples (5 ml) were collected from patients in the CHB and LC groups during outpatient visits and from the HBV-HCC group before surgical resection. Samples were centrifuged at 4000 rpm for 5 minutes, and the serum supernatant was stored at -40°C for subsequent analysis.

### Serum ESPL1 levels detection

Serum ESPL1 levels were measured using an Enzyme-Linked Immunosorbent Assay (ELISA) kit (Nova Lifetech Inc., Catalog No.: ELI-48263h) according to the manufacturer’s instructions. The procedure included standard and sample preparation, incubation, washing, addition of reagents, and optical density measurement. The absorbance (OD value) of each sample was quantified using a microplate reader (BioRad, Co., Ltd., USA) at a wavelength of 450 nm. Three replicate wells were utilized for each sample, and the mean value was determined. The concentration of serum ESPL1 in the unknown samples was calculated based on the standard curve (14).

### Statistical analysis

Data from the study was analyzed and visualized using SPSS version 26.0 (IBM Corp, Armonk, NY, USA) and R software version 3.6.2. Categorical variables were presented as counts (percentages), while continuous variables were described using median values with interquartile ranges. Spearman correlation tests were used to assess relationships between variables and to construct correlation heat maps. Categorical variables, such as sex and antiviral therapy, were converted into ordinal data before performing the analysis, in accordance with the requirements for Spearman’s rank correlation. Differences between groups for categorical variables were evaluated using the Chi-square test, whereas differences for continuous variables were assessed using the Kruskal-Wallis non-parametric test. Receiver Operating Characteristic (ROC) curves were plotted to evaluate the diagnostic capabilities of ESPL1, AFP, and PIVKA-II for HBV-HCC, and the areas under the curve (AUC) for these markers were compared. ROC analysis was used to determine the optimal cut-off threshold of ESPL1 for diagnosing HBV-HCC (19). A p-value of less than 0.05 was considered statistically significant.

## Results

### Patient characteristics

This study included a total of 266 patients, with clinical characteristics as shown in **Table 1**. The cohort comprised 121 patients with CHB, 98 with LC, and 47 with HBV-HCC, all of whom had HCC lesions smaller than 3 cm in diameter. The median age of patients in the CHB group, LC group, and HBV-HCC group showed an increasing trend, and there was a statistical difference in pairwise comparisons. The study enrolled 204 males (76.7%) and 62 females (23.3%), with the highest proportion of males in the HBV-HCC group (41 [87.2%]), although no significant differences were found between groups in pairwise comparisons. The proportion of HBV DNA positive patients was significantly higher in the CHB and HBV-HCC groups compared to the LC group, with no significant difference between CHB and HBV-HCC (CHB vs. LC, P=0.026; CHB vs. HBV-HCC, P=0.241; LC vs. HBV-HCC, P=0.002). No significant differences were observed in the proportion of HBeAg positive patients across the three groups. Serum ALT levels were significantly higher in the HBV-HCC group compared to the LC and CHB groups (P<0.05), but there was no significant difference between the LC and CHB groups (CHB vs. LC, P=0.057; CHB vs. HBV-HCC, P<0.001; LC vs. HBV-HCC, P=0.034). Regarding antiviral treatment, the highest proportion of patients on non-first-line nucleotide analogs (NAs) or combination therapy was observed in the HBV-HCC group, showing statistically significant differences, whereas no significant differences were noted between the LC and CHB groups (CHB vs. LC, P=0.202; CHB vs. HBV-HCC, P<0.001; LC vs. HBV-HCC, P<0.001).

**Table 1:** Clinical characteristics of the 266 patients included in the study.

Variable	CHB (n=121)	LC (n=98)	HBV-HCC (n=47)	*P* value
Age (years)	39 (34,47)	47 (40,55)	52 (44,60)	<0.001
Sex, n (%)				
Male	86(71.1)	77(78.6)	41(87.2)	0.072
Female	35(28.9)	21(21.4)	6(12.8)	
HBV DNA positive, n (%)	32 (28.9)	15 (15.3)	18(38.3)	0.006
HBeAg positive, n (%)	37 (30.6)	30 (30.6)	16 (34.0)	0.898
ALT (U/L)	24(17,35)	27(21,38)	32(26,48)	0.001
ESPL1 (ng/L)	224.6 (177.5,300.9)	285.8(228.9,324.9)	440.4(325.4,565.9)	<0.001
PIVKA-II (mAu/ml)	26.2(22.8,31.8)	29.4(22.7,34.2)	62.7(36.0,301.1)	<0.001
AFP (ng/ml)	2.5(1.8,3.2)	2.7(2.0,4.3)	6.2(3.0,118.2)	<0.001
Antiviral treatment drugs, n (%)				
Non-first-line NAs	3(2.5)	0	20(42.6)	<0.001
First-line NAs	77(63.6)	73(74.5)	20(42.6)	
Mixed medications	41(33.9)	25(25.5)	7(14.9)	
No antiviral treatment	2(1.7)	0	19(40.4)	
PIVKA-II positive, n (%)	11 (9.1)	12 (12.2)	32 (68.1)	<0.001
AFP positive, n (%)	1 (0.8)	0	7 (14.9)	<0.001

Note: ALT refers to Alanine Aminotransferase; ESPL1 is separase (extra spindle pole bodies like 1) phosphatase; AFP denotes Alpha-Fetoprotein. For antiviral therapy, Entecavir and Tenofovir are defined as first-line antiviral drugs. A PIVKA-II level >40 mAu/ml is considered positive, and an AFP level >400 ng/ml is defined as positive.

P-values for pairwise comparisons:

Age: CHB vs. LC, P<0.001; CHB vs. HBV-HCC, P<0.001; LC vs. HBV-HCC, P=0.039.

ALT: CHB vs. LC, P=0.057; CHB vs. HBV-HCC, P<0.001; LC vs. HBV-HCC, P=0.034.

Antiviral treatment drugs: CHB vs. LC, P=0.202; CHB vs. HBV-HCC, P<0.001; LC vs. HBV-HCC, P<0.001.

PIVKA-II positive: CHB vs. LC, P=0.295; CHB vs. HBV-HCC, P<0.001; LC vs. HBV-HCC, P<0.001.

AFP positive: CHB vs. LC, P=1; CHB vs. HBV-HCC, P=0.001; LC vs. HBV-HCC, P<0.001.

HBV DNA positive: CHB vs. LC, P=0.026; CHB vs. HBV-HCC, P=0.241; LC vs. HBV-HCC, P=0.002.

### Trends in serum ESPL1, PIVKA-II, and AFP levels across different disease states

**Figure 1:**
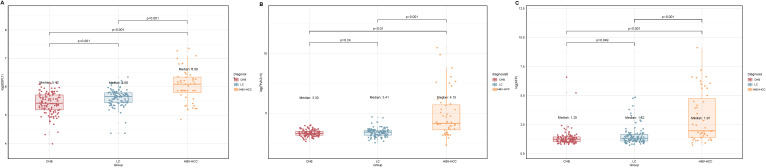
Log-transformed serum ESPL1, PIVKA-II, and AFP levels in different disease states. The box plots show the log-transformed values of serum ESPL1, PIVKA-II, and AFP levels in the CHB, LC, and HBV-HCC groups. The actual median serum levels for each group are provided in parentheses. **(A)** Serum ESPL1 levels showed an increasing trend from CHB to LC and HBV-HCC (median log-values: 5.42, 5.66, and 6.09), with actual median concentrations of 224.6 ng/L, 285.8 ng/L, and 440.4 ng/L, respectively. ESPL1 levels were significantly higher in the LC group than in the CHB group (P < 0.001), and significantly elevated in the HBV-HCC group compared to both CHB and LC groups (P < 0.001). **(B)** Serum PIVKA-II levels remained relatively stable between the CHB and LC groups (median log-values: 3.30 vs. 3.41; actual medians: 26.2 vs. 29.4 mAu/ml, P = 0.24) but showed a marked increase in the HBV-HCC group (median log-value: 4.15; actual median: 62.7 mAu/ml, P < 0.001). **(C)** Serum AFP levels demonstrated a progressive increase across the CHB, LC, and HBV-HCC groups (median log-values: 1.35, 1.52, and 1.97), with actual median concentrations of 2.5 ng/ml, 2.7 ng/ml, and 6.2 ng/ml, respectively. AFP levels were significantly higher in the HBV-HCC group compared to the CHB and LC groups (P < 0.001), with a significant difference also observed between the CHB and LC groups (P = 0.049). Statistical comparisons between the groups were performed using the Kruskal-Wallis’s test, with p-values indicated above the plots.

Serum ESPL1 levels showed an increasing trend across the CHB, LC, and HBV-HCC groups, with levels at 224.6 ng/L, 285.8 ng/L, and 440.4 ng/L respectively. Specifically, the ESPL1 levels in the LC group were significantly higher than those in the CHB group (P < 0.05), and the ESPL1 levels in the HBV-HCC group were significantly higher than those in the CHB and LC group (P < 0.05), as illustrated in **Figure 1**.

Serum PIVKA-II levels among CHB, LC, and HBV-HCC groups were 26.2 mAu/ml, 29.4 mAu/ml, and 62.7 mAu/ml, respectively. Levels in the HBV-HCC group were significantly higher than those in the CHB and LC groups, with a statistically significant difference (P<0.05). There was no significant difference in serum PIVKA-II levels between the CHB and LC groups (P>0.05), as shown in **Figure 1**.

Serum AFP levels among patients in the CHB, LC, and HBV-HCC groups were 2.5 ng/ml, 2.7 ng/ml, and 6.2 ng/ml, respectively. Levels in the HBV-HCC group were significantly higher compared to those in the CHB and LC groups, with a statistically significant difference (P<0.05). There was significant difference in serum AFP levels between the CHB and LC groups, as illustrated in **Figure 1**.

### Relationship between ESPL1, PIVKA-II, AFP levels and clinical characteristics

**Figure 2:**
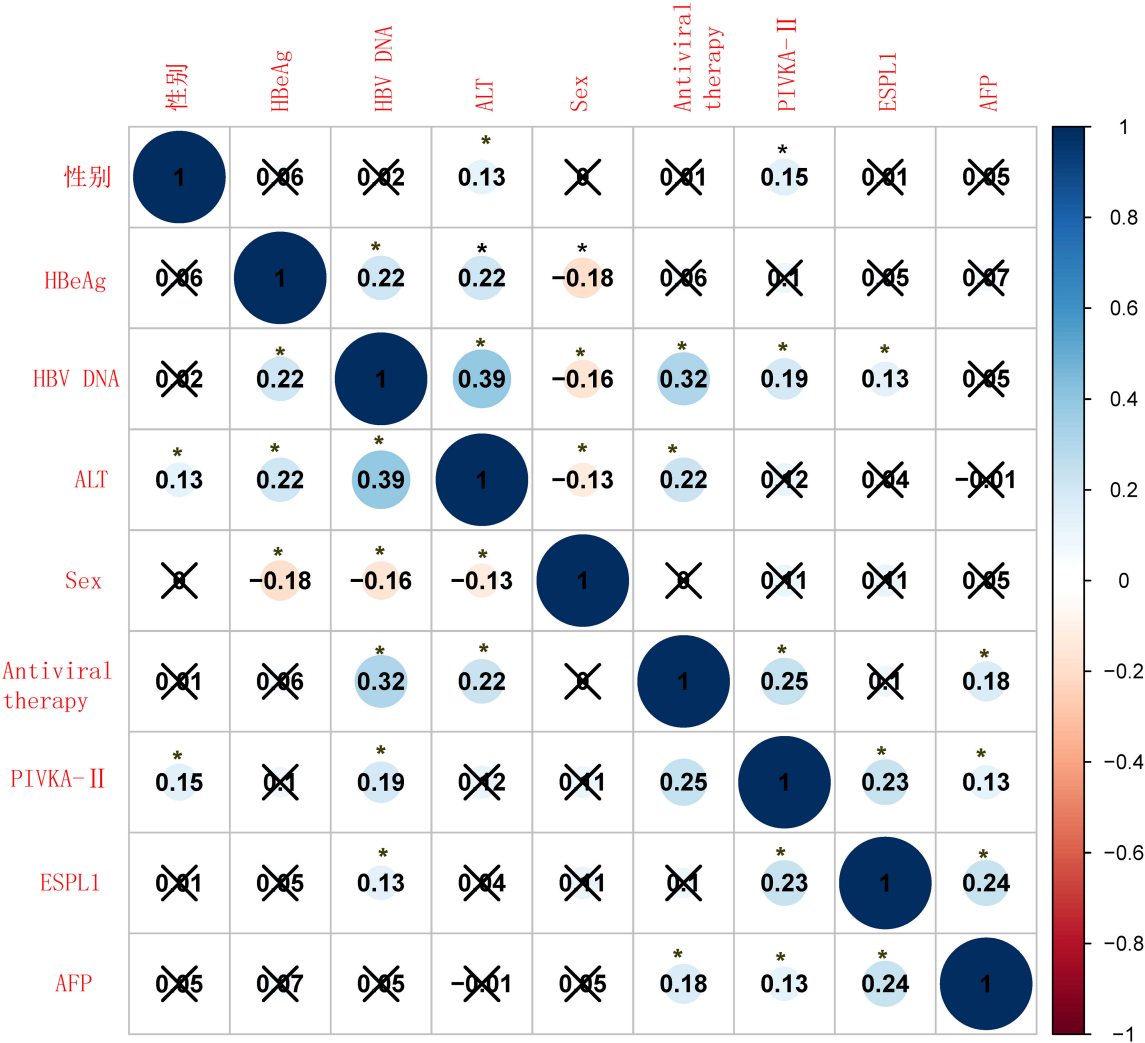
Correlation heatmap of clinical characteristics and biomarkers. The heatmap shows the Spearman correlation coefficients between various clinical variables and biomarkers. Positive correlations are shown in blue, and negative correlations are shown in red. The size of the circles represents the strength of the correlation, with larger circles indicating stronger correlations. Statistical significance is indicated by an asterisk (*), with p-values less than 0.05, while a “×” indicates the absence of a statistically significant difference.

As shown in **Figure 2**, serum levels of ESPL1, PIVKA-II, and AFP are not significantly affected by the patients’ clinical characteristics, and no strong correlations were observed between these markers. Specifically, serum ESPL1 levels were weakly positively correlated with HBV DNA status (P<0.05, r=0.13), but did not correlate with patient age, HBeAg status, antiviral medication, ALT levels, or gender (P>0.05). Serum AFP levels showed a weak positive correlation with antiviral medication use (P<0.05, r=0.18), but no significant correlation with HBV DNA status, HBeAg status, age, ALT levels, or gender (P>0.05). Serum PIVKA-II levels were weakly positively correlated with gender, HBV DNA status, and antiviral medication use (P<0.05, r values of 0.15, 0.19, and 0.25, respectively), but showed no significant correlation with patient age, HBeAg status, or ALT levels (P>0.05).

### The early diagnostic value of ESPL1, PIVKA-II and AFP for HBV- HCC

**Figure 3:**
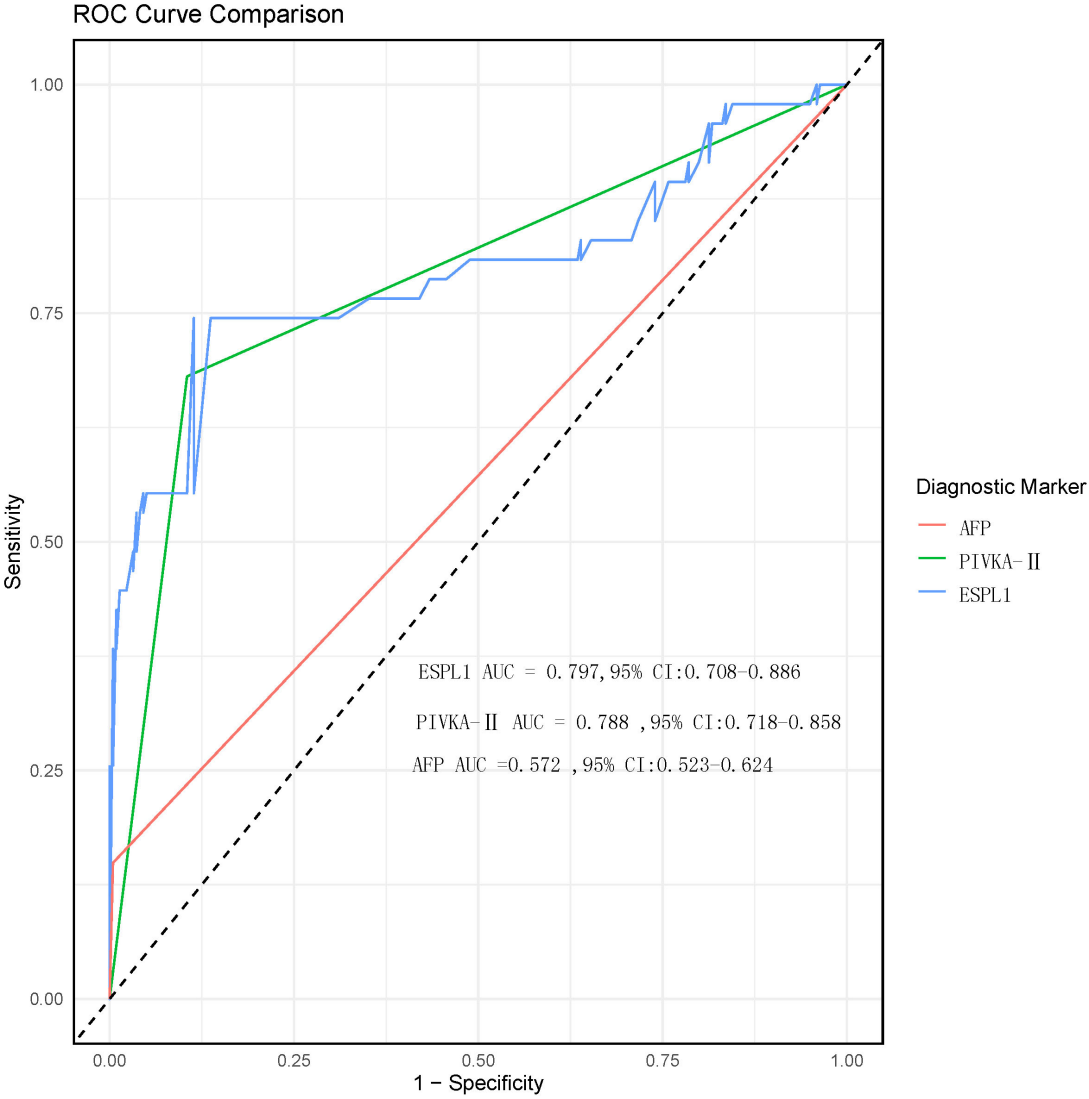
ROC curve comparison of diagnostic biomarkers for HBV-HCC. The ROC curves for ESPL1 (blue), PIVKA-II (green), and AFP (red) are shown to assess their diagnostic performance for HBV-HCC. Although the AUC values for ESPL1 and PIVKA-II are higher than those for AFP, the comparison between the three biomarkers did not show statistically significant differences.

According to the diagnostic standard values for AFP and PIVKA-II (AFP >400 ng/ml, PIVKA-II >40 mAu/ml), they are categorized as positive or negative. The ROC curve analysis results for serum levels of ESPL1, PIVKA-II, and AFP in diagnosing HBV-HCC are shown in **Figure 3**. The ROC curve identifies the optimal diagnostic cutoff for ESPL1 as 345.7 ng/L. The AUC from highest to lowest are ESPL1: 0.797 (95% CI: [0.708-0.886]), PIVKA-II: 0.788 (95% CI: [0.718-0.858]), and AFP: 0.572 (95% CI: [0.523-0.624]), with no significant differences between each pair (P>0.05). According to the ROC analysis, the specificity of all three markers for diagnosing HBV-HCC is similar, PIVKA-II has the lowest sensitivity, and ESPL1 has the highest Youden index. The predictive value of ESPL1, PIVKA-II, and AFP in the early diagnosis of HBV-HCC is detailed in **Table 2**.

**Table 2:** Application of serum ESPL1, PIVKA-II, and AFP in the diagnosis of HBV-HCC.

Diagnostic markers	AUC (95%CI)	Specificity	Sensitivity	Positive predictive value	Negative predictive value	Youden index
PIVKA-II	0.788 (0.718-0.858)	93%	58%	68%	89%	0.51
ESPL1	0.797 (0.708-0.886 )	89%	74%	74%	89%	0.63
AFP	0.572 (0.523-0.624)	99%	15%	88%	85%	0.14

**Figure 4:**
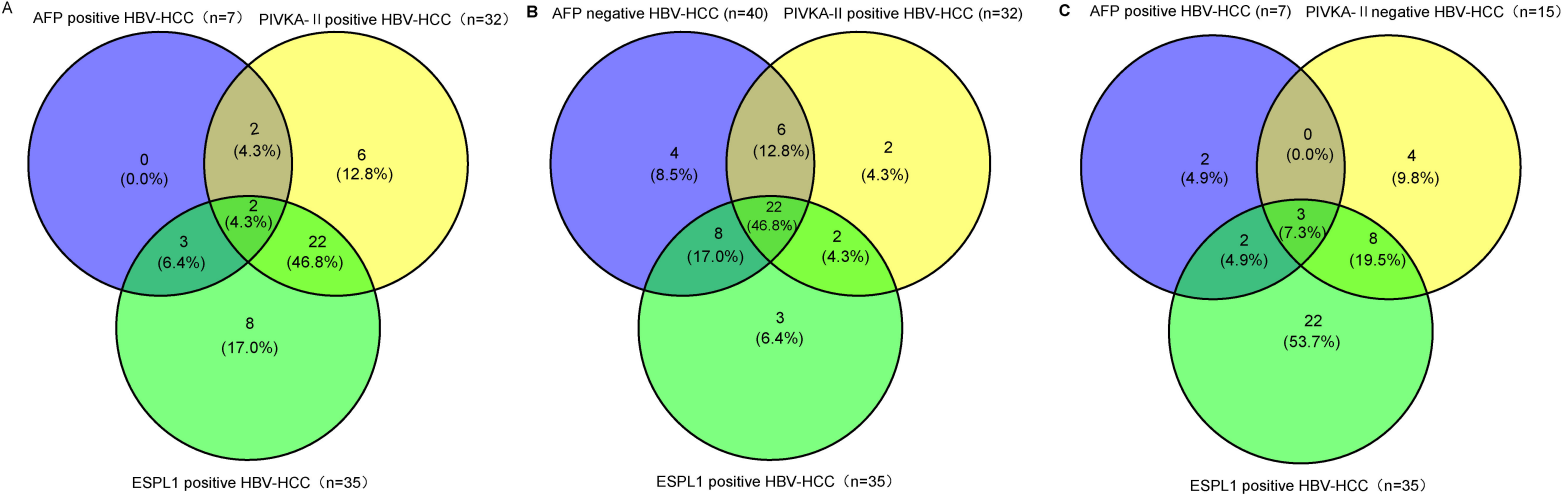
The Venn diagrams depict the distribution of ESPL1, PIVKA-II, and AFP positivity in HBV-HCC patients, emphasizing the superior positivity rate of ESPL1 in different subgroups. **(A)** Among AFP-positive HBV-HCC patients, ESPL1 demonstrated a higher detection rate than PIVKA-II. Notably, no patients were exclusively AFP-positive, while ESPL1 alone identified a significant proportion of cases, reinforcing its diagnostic value beyond AFP. **(B)** In AFP-negative HBV-HCC patients, ESPL1 exhibited the highest positivity rate, surpassing PIVKA-II. This suggests that ESPL1 may serve as a more effective diagnostic biomarker in AFP-negative cases, where conventional markers often fail. **(C)** Among PIVKA-II-negative HBV-HCC patients, ESPL1 positivity remained markedly higher than AFP, further confirming its diagnostic potential even in cases where PIVKA-II is absent. Together, these findings highlight that ESPL1 outperforms AFP and PIVKA-II in HBV-HCC detection, reinforcing its potential role as a more reliable biomarker, particularly in PIVKA-II and AFP-negative cases.

In patients with hepatitis and cirrhosis who were positive for AFP, no ESPL1 positivity was detected. Conversely, among patients with hepatitis and cirrhosis who were positive for PIVKA-II, 2 out of 23 (8.7%) exhibited ESPL1 positivity. Additionally, four HBV-HCC patients tested negative for ESPL1, PIVKA-II, and AFP, whereas two patients were positive for all three biomarkers. As illustrated in **Figure 4**, among the 47 confirmed HBV-HCC patients, the positive rate of ESPL1 (74.5%) was higher than that of PIVKA-II (68.1%) and AFP (6.9%). Furthermore, in HBV-HCC patients, irrespective of AFP status (**Figures 4**), the positive rate of ESPL1 surpassed that of PIVKA-II. Similarly, in HBV-HCC patients, irrespective of PIVKA-II status (**Figures 4**), the positive rate of ESPL1 exceeded that of AFP.

**Figure 5:**
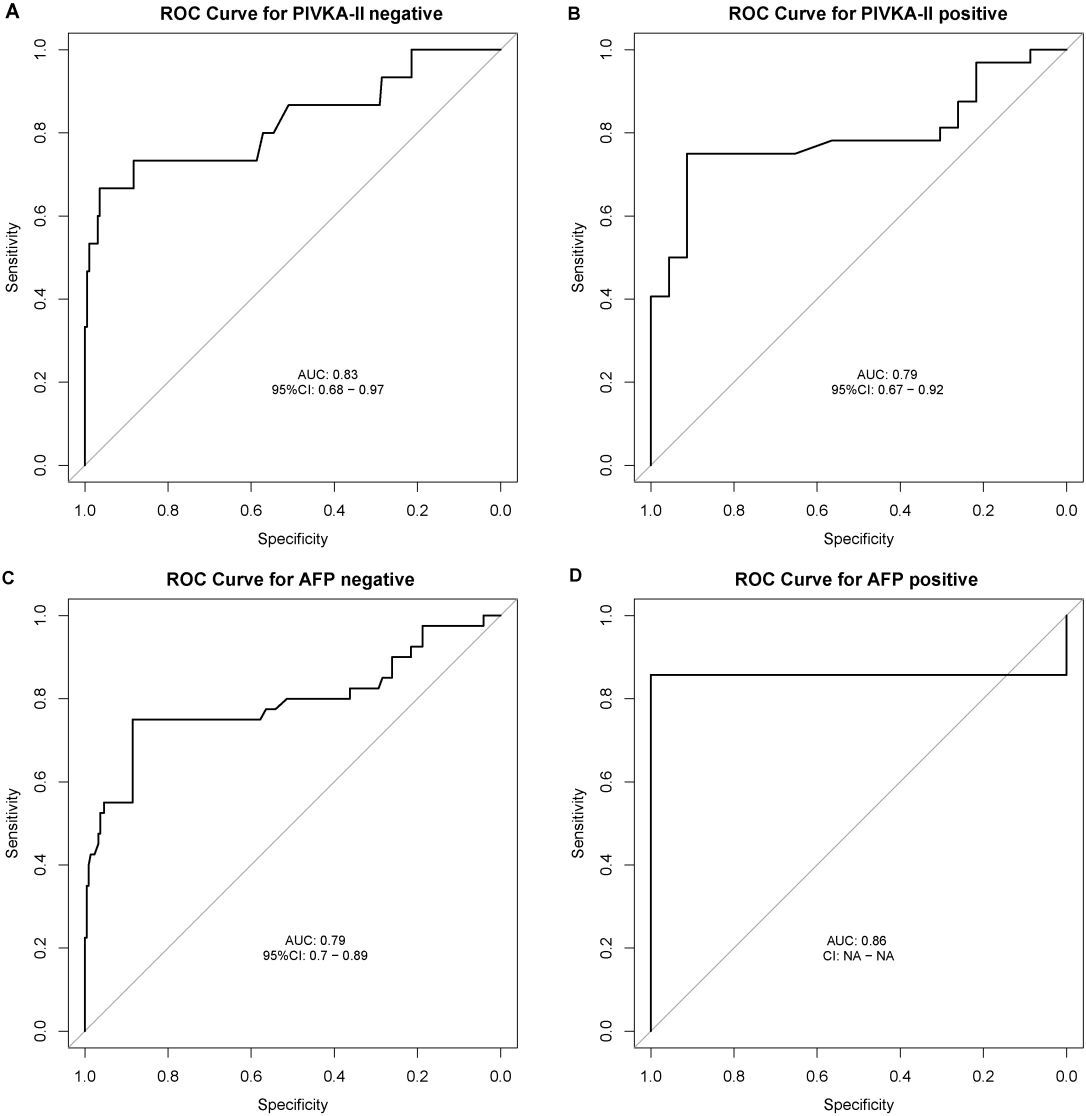
Receiver Operating Characteristic (ROC) Curves for ESPL1 in Diagnosing HBV-HCC Stratified by AFP and PIVKA-II Statuses. The ROC curves illustrate the diagnostic performance of ESPL1 for HBV-HCC across different biomarker subgroups. **(A)** In patients negative for PIVKA-II, ESPL1 achieves an AUC of 0.83 (95% CI: 0.68–0.97), demonstrating strong discriminatory ability. **(B)** Among PIVKA-II positive individuals, ESPL1 maintains a comparable AUC of 0.79 (95% CI: 0.67–0.92). **(C)** Similarly, in AFP-negative patients, ESPL1 exhibits an AUC of 0.79 (95% CI: 0.70–0.89). **(D)** The highest AUC of 0.86 is observed in AFP-positive patients, reinforcing its diagnostic potential. These findings suggest that ESPL1 remains a robust biomarker for HBV-HCC diagnosis regardless of AFP and PIVKA-II statuses.

Regardless of AFP and PIVKA-II statuses, the AUC for ESPL1 in diagnosing HBV-HCC remains consistent. For patients negative for both AFP and PIVKA-II, the AUC values for ESPL1 in diagnosing HBV-HCC are 0.79 and 0.83, respectively. The ROC curves demonstrating these findings are shown in **Figures 5A–D**.

## Discussion

Our research demonstrates that serum ESPL1 testing offers superior early diagnostic value for HBV-HCC compared to AFP and PIVKA-II, especially in patients who are negative for both AFP and PIVKA-II. Most patients (over 70%) with small HBV-HCC who are negative for both AFP and PIVKA-II show positive ESPL1 levels. In patients with CHB and LC who are positive for AFP, no ESPL1 positivity was observed, while in those positive for PIVKA-II, ESPL1 positivity was found in 2 (8.7%) cases. The AUC for ESPL1 in diagnosing HBV-HCC remains consistent regardless of the status of AFP and PIVKA-II. Therefore, testing for ESPL1 in chronic HBV-infected patients with liver nodules can aid in the differential diagnosis of HBV-HCC.

Contemporary medicine has made significant advances in diagnosing and treating HBV-HCC, yet finding an efficient, sensitive, convenient, cost-effective, and timely method for early HBV-HCC screening and diagnosis remains a challenge for clinicians (20). Currently, the diagnosis of HBV-HCC heavily relies on imaging or histopathology, such as ultrasound, contrast-enhanced ultrasound, CT scans, liver MRI, or liver biopsy. However, the accuracy of imaging depends largely on the quality of the equipment and the experience of the examiner. Techniques like ultrasound, CT, and MRI have limitations in distinguishing the malignancy of small nodules, and their high costs restrict their widespread clinical screening application, thus limiting their value for early diagnosis of HBV-HCC. While imaging-based diagnostics are recommended by guidelines, histopathology remains the gold standard for HBV-HCC diagnosis. Nevertheless, biopsy carries risks such as tumor cell spread along the needle track, potentially leading to extensive liver metastasis, and the technical demands of biopsy make it less suitable for screening purposes. Compared to these methods, serum tumor marker testing is easier to perform, cost-effective, repeatable, and sufficiently accurate, making it more favorable for disease screening and early diagnostic alerts. The search for new biomarkers that accurately reflect the natural history or progression stages of chronic HBV infection, closely monitoring disease progression, and early detection of HBV-HCC continues to pose a significant clinical challenge (10).

To explore the value of serum ESPL1 levels in early warning and diagnosis of HBV-HCC, we included patients with pathologically confirmed small HBV-HCC. Our findings indicate that the areas under the ROC curves for ESPL1, AFP, and PIVKA-II in diagnosing HBV-HCC are similar, with no statistically significant differences; the optimal diagnostic threshold for ESPL1 was identified as 345.7 ng/L. Unlike AFP and PIVKA-II, ESPL1 levels progressively increased across the CHB, LC, and HBV-HCC groups with substantial changes, specifically from 224.6 ng/L to 285.8 ng/L to 440.4 ng/L. In contrast, AFP and PIVKA-II levels showed no statistical differences between the CHB and LC groups, and AFP levels were not notably high across the groups, with a median of only 10.5 ng/ml in the HBV-HCC group, much lower than the guideline-recommended 400 ng/ml (10). In addition, although PIVKA-II levels reached the guideline-recommended positive threshold in the HCC group, they did not show a significant increase with the progression from CHB to LC. This suggests that PIVKA-II levels do not provide a useful early warning before disease progression to HCC in CHB patients. Our earlier research also confirmed that in HBV-HCC patients, serum AFP and PIVKA-II levels increase with tumor diameter but are not associated with histological grading. Serum ESPL1 levels are significantly higher in HBV-HCC patients compared to those with non-HBV-HCC and correlate positively with histological grade; tumor size does not strictly correlate with malignancy, indicating that serum ESPL1 levels can reflect the malignancy of HBV-HCC and aid in the differential diagnosis of liver nodules in HBV-infected individuals (12). Song et al. have also detected high expression of the ESPL1 gene in liver cancer tissues (21). Therefore, the determination of serum ESPL1 levels is not only helpful for the diagnosis of HBV-HCC but may also offer early warning information for the development of HBV-HCC. In our study, a small subset of patients with ESPL1 levels exceeding 345.7 ng/L in the CHB or LC groups eventually developed liver nodules. This suggests that elevated ESPL1 levels could serve as an early indicator for the potential development of HBV-HCC. Continual monitoring of patients with ESPL1 levels above this threshold, particularly if levels show a gradual increase, could provide valuable early warning, enabling earlier intervention and more effective management of at-risk individuals.

Moreover, our research revealed a lack of statistically significant association between ESPL1 levels and variables such as antiviral medication usage, tumor size, HBV DNA and HBeAg status, age, gender, or ALT levels. These findings indicate that serum ESPL1 levels may offer consistent and dependable diagnostic outcomes across diverse clinical scenarios, enhancing the overall diagnostic capacity for HBV-HCC. Therefore, while elevated serum AFP and PIVKA-II levels often indicate existing HCC, dynamic monitoring of ESPL1 levels, particularly in patients with values exceeding 345.7 ng/L, may provide an effective early warning for the onset of HBV-HCC. This could offer a clinical basis for personalized screening strategies, especially for high-risk populations such as those with CHB or LC, enabling timely intervention and more effective management.

More importantly, an ideal serum marker for early warning and diagnosis of HBV-HCC should exhibit high sensitivity and specificity. Biomarkers with a specificity exceeding 80% are considered to have significant diagnostic value (22). Currently, AFP and PIVKA-II are widely accepted as effective diagnostic markers for HBV-HCC. Our research indicates that ESPL1 demonstrates a diagnostic specificity of 89%, comparable to AFP and PIVKA-II, with a significantly higher sensitivity. Therefore, it is reasonable to suggest that ESPL1 may be more suitable than AFP and PIVKA-II for early warning and diagnosis of HBV-HCC. We also found that a significant proportion of patients with small HBV-HCC who tested negative for both AFP and PIVKA-II exhibited elevated levels of ESPL1. This suggests that ESPL1 may enhance the diagnostic capabilities of AFP and PIVKA-II in the early detection of HBV-HCC, thereby improving the overall rate of early detection. Furthermore, the ROC curve analysis demonstrated that serum ESPL1 levels maintained consistent diagnostic efficacy in patients with either negative or positive AFP and PIVKA-II results. These findings underscore the clinical significance of serum ESPL1 protein levels in HBV-HCC diagnosis, particularly in individuals with negative AFP and PIVKA-II results.

Our study also has certain limitations. Firstly, it is a single-center study, lacking the broader validation that multi-center, large-sample studies could provide to further confirm the value of ESPL1 in the early warning and diagnosis of HBV-HCC. Secondly, our study is retrospective, which could introduce selection bias. However, by comparing the levels of ESPL1, AFP, and PIVKA-II in peripheral blood at the same time point for the same patients, we have added credibility to our results. Moving forward, we plan to conduct longitudinal studies to compare changes in these serum markers before and after the onset of HBV-HCC in the same patients, further substantiating the role of ESPL1 in the early warning and diagnosis of HBV-HCC.

## Conclusion

ESPL1 holds promise as a novel biomarker for chronic HBV infection, reflecting its natural history and stages of disease progression. Monitoring changes in serum ESPL1 levels could serve as an effective early warning for the development of HBV- HCC. This approach supports the creation of personalized screening strategies for the treatment of individuals with chronic HBV, providing a clinical basis for targeted interventions.

## Data Availability

The datasets used and/or analyzed during the current study are available from the corresponding author upon reasonable request.

## References

[1] Rumgay H Arnold M Ferlay J Lesi O Cabasag CJ Vignat J. Global burden of primary liver cancer in 2020 and predictions to 2040. J Hepatol. (2022) 77:1598–606. doi: 10.1016/j.jhep.2022.08.021 PMC9670241 36208844

[2] Yang X Lan T Zhong H Zhang Z Xie H Li Y. To systematically evaluate and analyze the efficacy and safety of transcatheter arterial chemoembolization (TACE) in the treatment of primary liver cancer. J Healthc Eng. (2022) 2022:8223336. doi: 10.1155/2022/8223336 35356619 PMC8959991

[3] Fernández-Barrena MG Arechederra M Colyn L Berasain C Avila MA. Epigenetics in hepatocellular carcinoma development and therapy: The tip of the iceberg. JHEP Rep. (2020) 2:100167. doi: 10.1016/j.jhepr.2020.100167 33134907 PMC7585149

[4] Hatanaka T Yata Y Naganuma A Kakizaki S. Treatment strategy for intermediate-stage hepatocellular carcinoma: transarterial chemoembolization, systemic therapy, and conversion therapy. Cancers (Basel). (2023) 15(6):1798. doi: 10.3390/cancers15061798 36980684 PMC10046825

[5] Shi Y Wang Y Niu K Zhang W Lv Q Zhang Y. How CLSPN could demystify its prognostic value and potential molecular mechanism for hepatocellular carcinoma: A crosstalk study. Comput Biol Med. (2024) 172:108260. doi: 10.1016/j.compbiomed.2024.108260 38492457

[6] Shu H Li W Shang S Qin X Zhang S Liu Y. Diagnosis of AFP-negative early-stage hepatocellular carcinoma using Fuc-PON1. Discovery Med. (2017) 23:163–8. 28472609

[7] Piñero F Dirchwolf M Pessôa MG. Biomarkers in hepatocellular carcinoma: diagnosis, prognosis and treatment response assessment. Cells. (2020) 9(6):1370. doi: 10.3390/cells9061370 32492896 PMC7349517

[8] Kurokawa T Yamazaki S Mitsuka Y Moriguchi M Sugitani M Takayama T. Prediction of vascular invasion in hepatocellular carcinoma by next-generation des-r-carboxy prothrombin. Br J Cancer. (2016) 114:53–8. doi: 10.1038/bjc.2015.423 PMC4716541 26679378

[9] Chen H Zhang Y Li S Li N Chen Y Zhang B. Direct comparison of five serum biomarkers in early diagnosis of hepatocellular carcinoma. Cancer Manage Res. (2018) 10:1947–58. doi: 10.2147/CMAR.S167036 PMC6044429 30022853

[10] Chinese Society of Hepatology CMACSoID Chinese Medical Association. Guidelines for the prevention and treatment of chronic hepatitis B(version 2022). J Prac Hepatol. (2023) 26:457–78.

[11] Hu B Huang W Wang R Zang W Su M Li H. Li QQ et al: High Rate of Detection of Human ESPL1-HBV S Fusion Gene in Patients With HBV-related Liver Cancer: A Chinese Case-Control Study. Anticancer Res. (2020) 40:245–52. doi: 10.21873/anticanres.13946 31892573

[12] Hu B Wei LU Liang H Su M Wang R Su T. Correlation between serum ESPL1 and hepatitis B virus-related hepatocellular carcinoma histological grade: A chinese single-center case-control study. Anticancer Res. (2023) 43:3997–4005. doi: 10.21873/anticanres.16587 37648308

[13] Hu B Deng D Liang H Wang R Su M Wei L. Serum ESPL1 protein as an early warning biomarker for the initial occurrence and recurrence of hepatitis B virus-related hepatocellular carcinoma. Res Square. (2023). https://www.researchsquare.com/article/rs-3474605/v1. version 1.

[14] Wang R Zang W Hu B Deng D Ling X Zhou H. Serum ESPL1 can be used as a biomarker for patients with hepatitis B virus-related liver cancer: A chinese case-control study. Technol Cancer Res Treat. (2020) 19:1533033820980785. doi: 10.1177/1533033820980785 33308056 PMC7739072

[15] Nie Z Pu T Han Z Wang C Pan C Li P. Wang C et al: Extra Spindle Pole Bodies-Like 1 Serves as a Prognostic Biomarker and Promotes Lung Adenocarcinoma Metastasis. Front Oncol. (2022) 12:930647. doi: 10.3389/fonc.2022.930647 35814478 PMC9257280

[16] Yang X Miao G Wang Q Yu Q Hu Q Tan G. E2F1-mediated ESPL1 transcriptional activation predicts poor prognosis and promotes the proliferation of leiomyosarcoma. CytoJournal. (2025) 22:3. doi: 10.25259/Cytojournal_178_2024 39958881 PMC11829311

[17] Liu Z Lian X Zhang X Zhu Y Zhang W Wang J. ESPL1 is a novel prognostic biomarker associated with the Malignant features of glioma. Front Genet. (2021) 12:666106. doi: 10.3389/fgene.2021.666106 34512713 PMC8428966

[18] Yang Y Sheng Y Zheng J Ma A Chen S Lin J. Upregulation of ESPL1 is associated with poor prognostic outcomes in endometrial cancer. Biomarkers: Biochem Indic exposure response susceptibility to chemicals. (2024) 29:185–93. doi: 10.1080/1354750X.2024.2339288 38568742

[19] Akobeng AK. Understanding diagnostic tests 3: Receiver operating characteristic curves. Acta Paediatr. (2007) 96:644–7. doi: 10.4103/picr.PICR_87_18 17376185

[20] Nguyen HB Le XT Nguyen HH Vo TT Le MK Nguyen NT. Diagnostic Value of hTERT mRNA and in Combination With AFP, AFP-L3%, Des-γ-carboxyprothrombin for Screening of Hepatocellular Carcinoma in Liver Cirrhosis Patients HBV or HCV-Related. Cancer Inform. (2022) 21:11769351221100730. doi: 10.1177/11769351221100730 35614962 PMC9125073

[21] Song R Huang J Yang C Li Y. ESPL1 is elevated in hepatocellular carcinoma and predicts prognosis. Int J Gen Med. (2022) 15:8381–98. doi: 10.2147/IJGM.S381188 PMC9717693 36465268

[22] Postuma RB Berg D Stern M Poewe W Olanow CW Oertel W. MDS clinical diagnostic criteria for Parkinson’s disease. Mov Disord. (2015) 30:1591–601. doi: 10.1002/mds.26424 26474316

